# Supplementary material for: Metabolomic Profiling Reveals Sex Specific Associations with Chronic Obstructive Pulmonary Disease and Emphysema
Source: Metabolites. 2021 Mar 11;11(3):161. doi: 10.3390/metabo11030161 (PMC7999201; doi:10.3390/metabo11030161)
Supplement: Supplementary file 1 [file metabolites-11-00161-s001.zip › Supplement - Metabolomic Sex Differences - 18Feb2021.docx]

**Supplementary Figures**


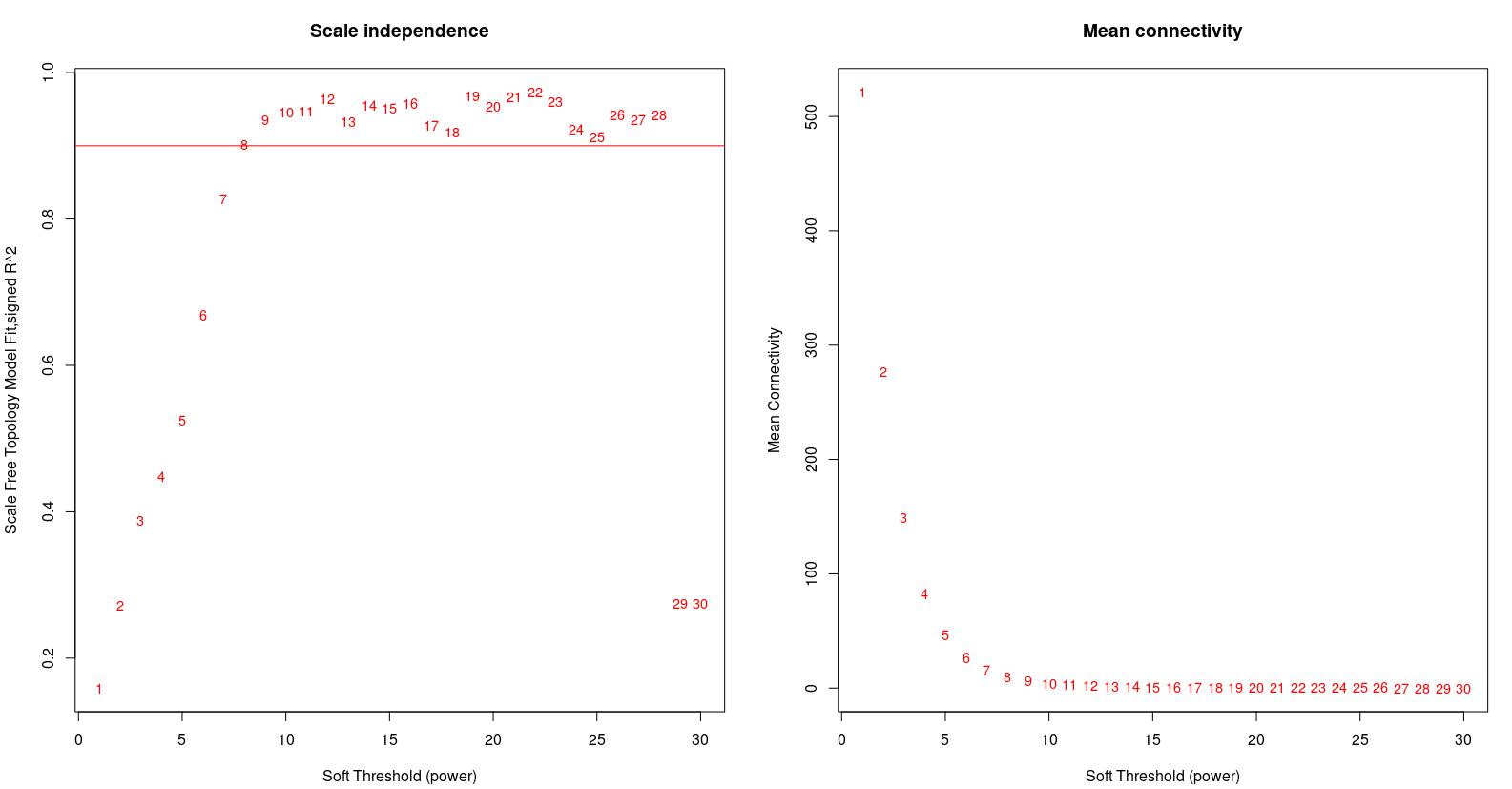


**Supplemental Figure 1. Scale Free Independence Plots for COPDGene**. **(A)** Shows scale free topology model fit (as assessed by the signed R^2^ of the model) for each soft threshold power between 1 and 30. The red line indicates the threshold for determining scale free topology. **(B)** Shows mean connectivity over all metabolites at each soft thresholding power.


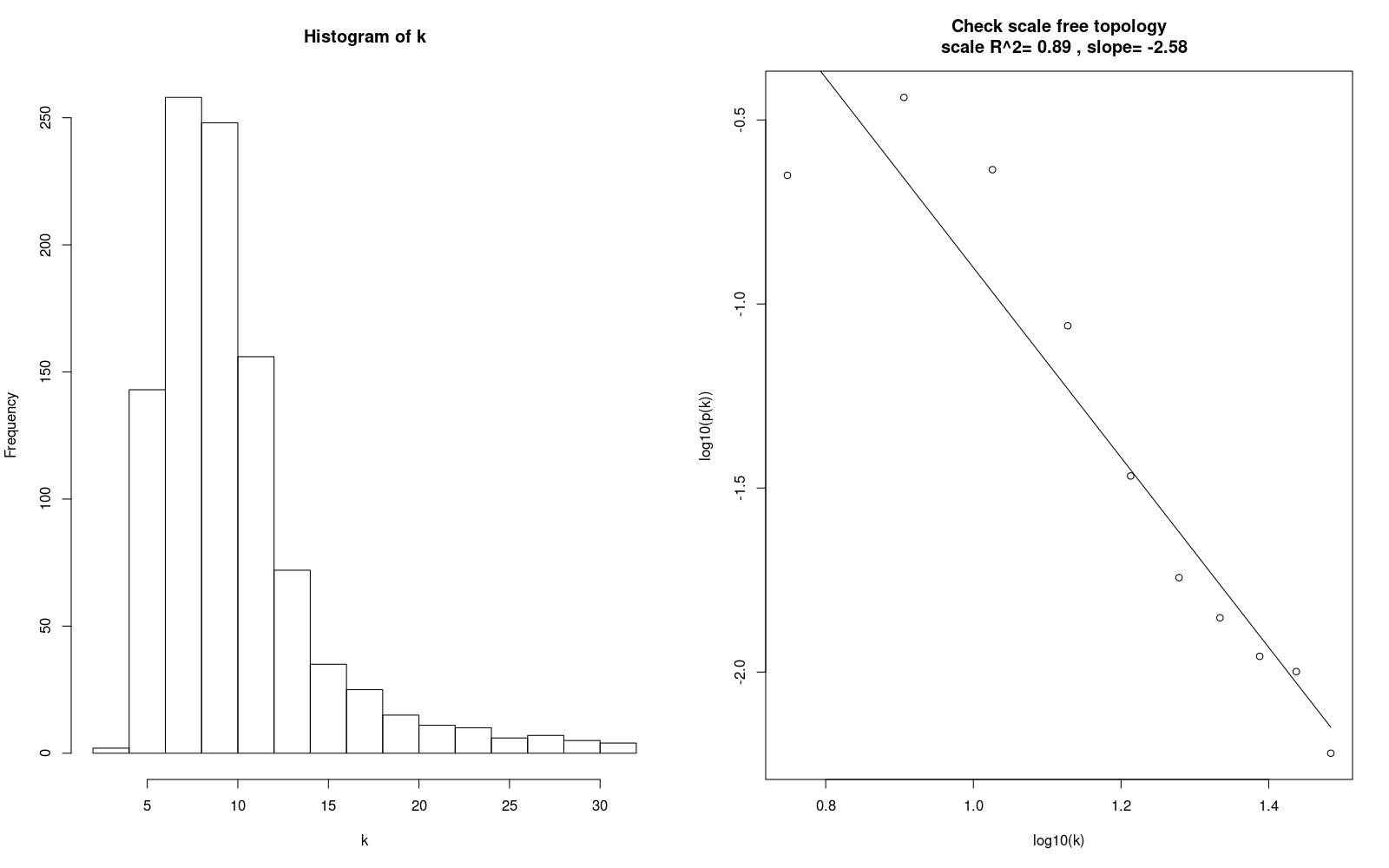
**Supplemental Figure 2**: Connectivity diributions to show scale-free topology of COPDGene. The frequency distribution of the connectivity (left) shows a large number of low connected metabolites and a small number of highly connected metabolites. The log-log plot shows an R2 (the scale-free topology index) of 0.89, which means the network is following the scale-free topology criterion.


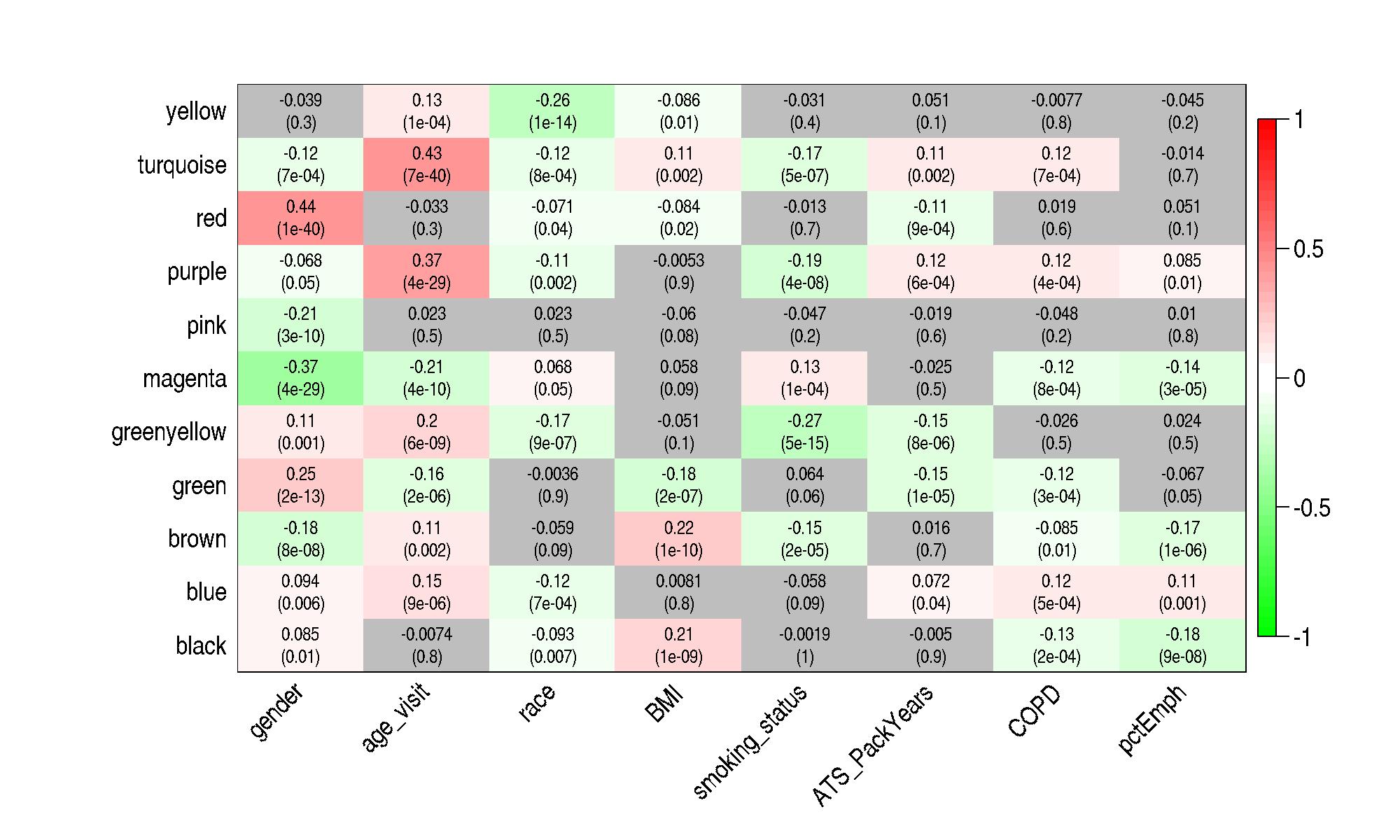
**Supplemental Figure S3.** Strength of correlation between *COPDGene* weighted gene co-expression network analysis (WGCNA) modules and clinical variables and outcomes. Heatmap of module/clinical variable correlation with green idicating positive correlations and red indicating negative. Cell text is Pearson correlation (p-value) between the first principal component representing the module and the corresponding variable.


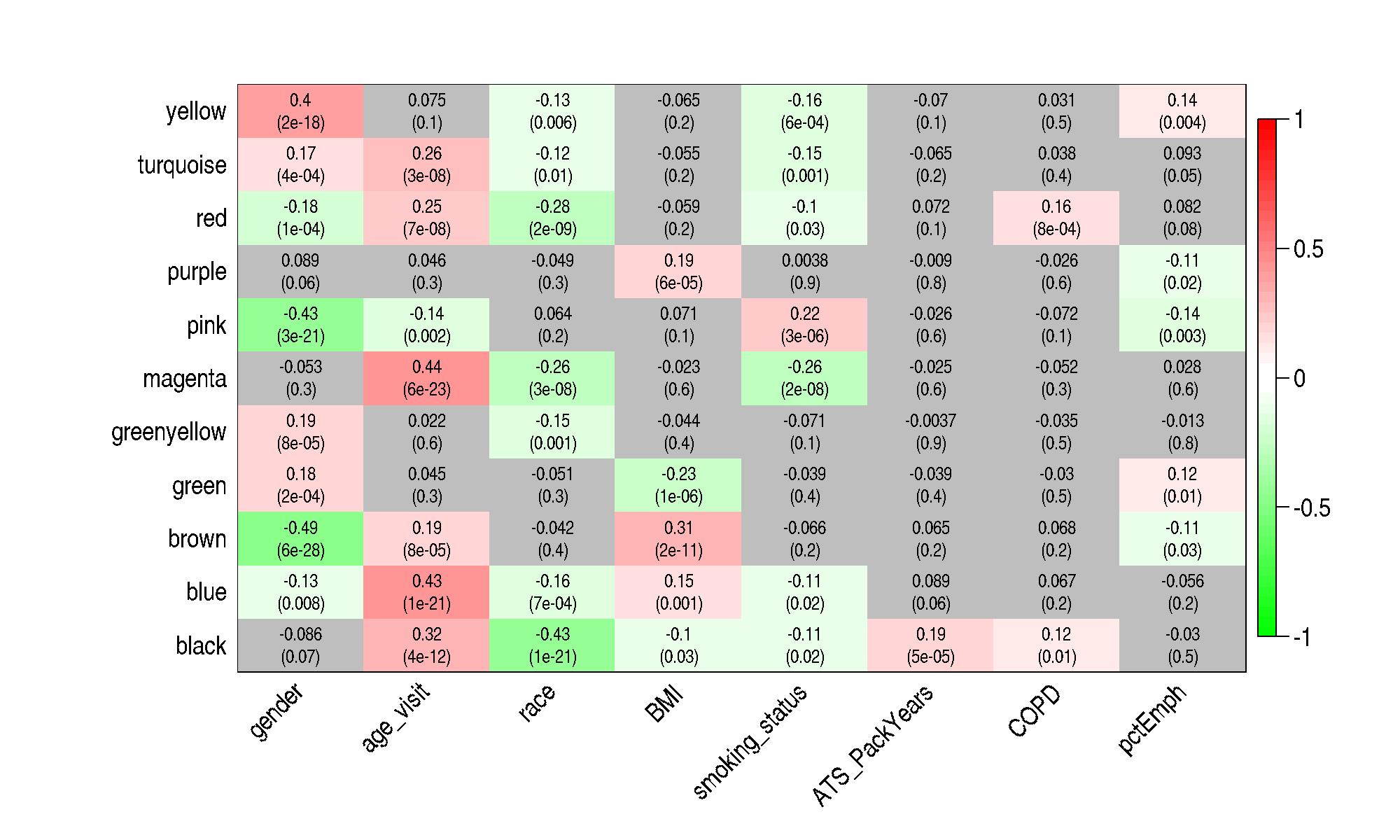
**Supplemental Figure S4.** Strength of correlation between *SPIROMICS* weighted gene co-expression network analysis (WGCNA) modules and clinical variables and outcomes. Heatmap of module/clinical variable correlation with green idicating positive correlations and red indicating negative. Cell text is Pearson correlation (p-value) between the first principal component representing the module and the corresponding variable.


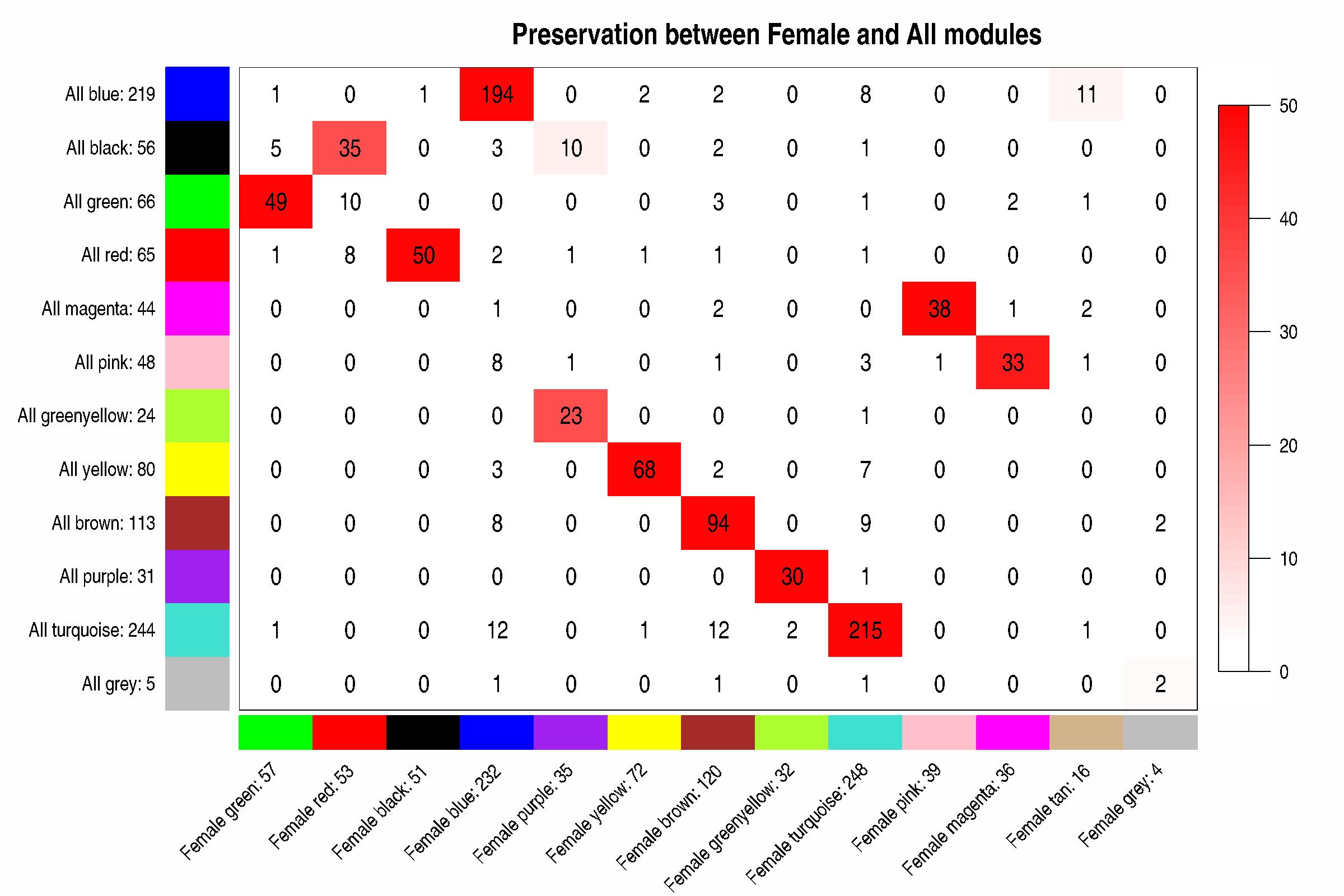


**Supplemental Figure S5: Correspondence of female set-specific modules and full cohort modules in COPDGene.** Each row of the table corresponds to one female set-specific module (labeled by color as well as text), and each column corresponds to one full cohort module. Numbers in the table indicate metabolite counts in the intersection of the corresponding modules. Coloring of the table encodes−log(p), with p being the Fisher’s exact test p-value for the overlap of the two modules. The stronger the red color, the more significant the overlap is.


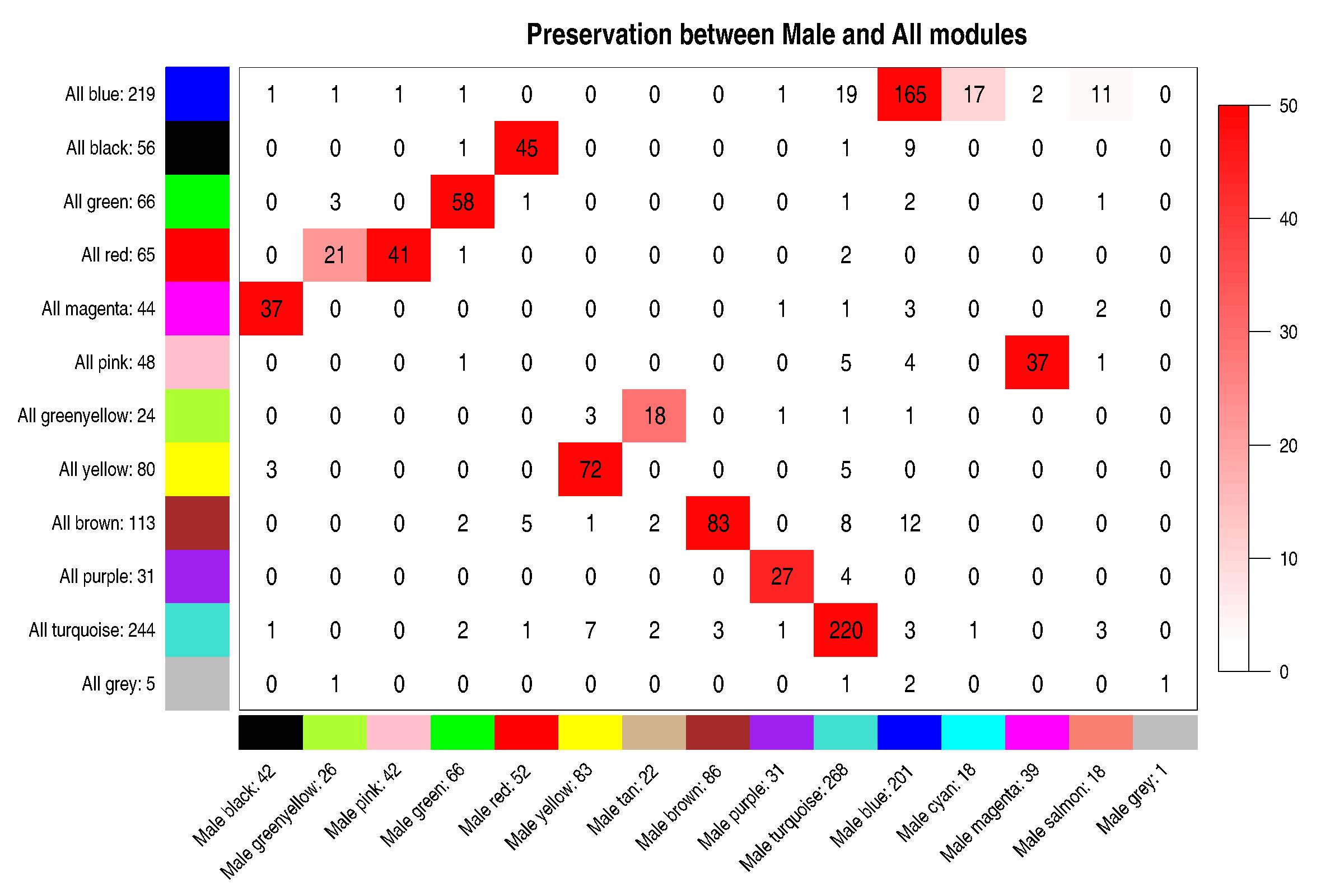


**Supplemental Figure S6: Correspondence of male set-specific modules and full cohort modules in *COPDGene*.** Each row of the table corresponds to one male set-specific module (labeled by color as well as text), and each column corresponds to one full cohort module. Numbers in the table indicate metabolite counts in the intersection of the corresponding modules. Coloring of the table encodes−log(p), with p being the Fisher’s exact test p-value for theverlap of the two modules. The stronger the red color, the more significant the overlap is.


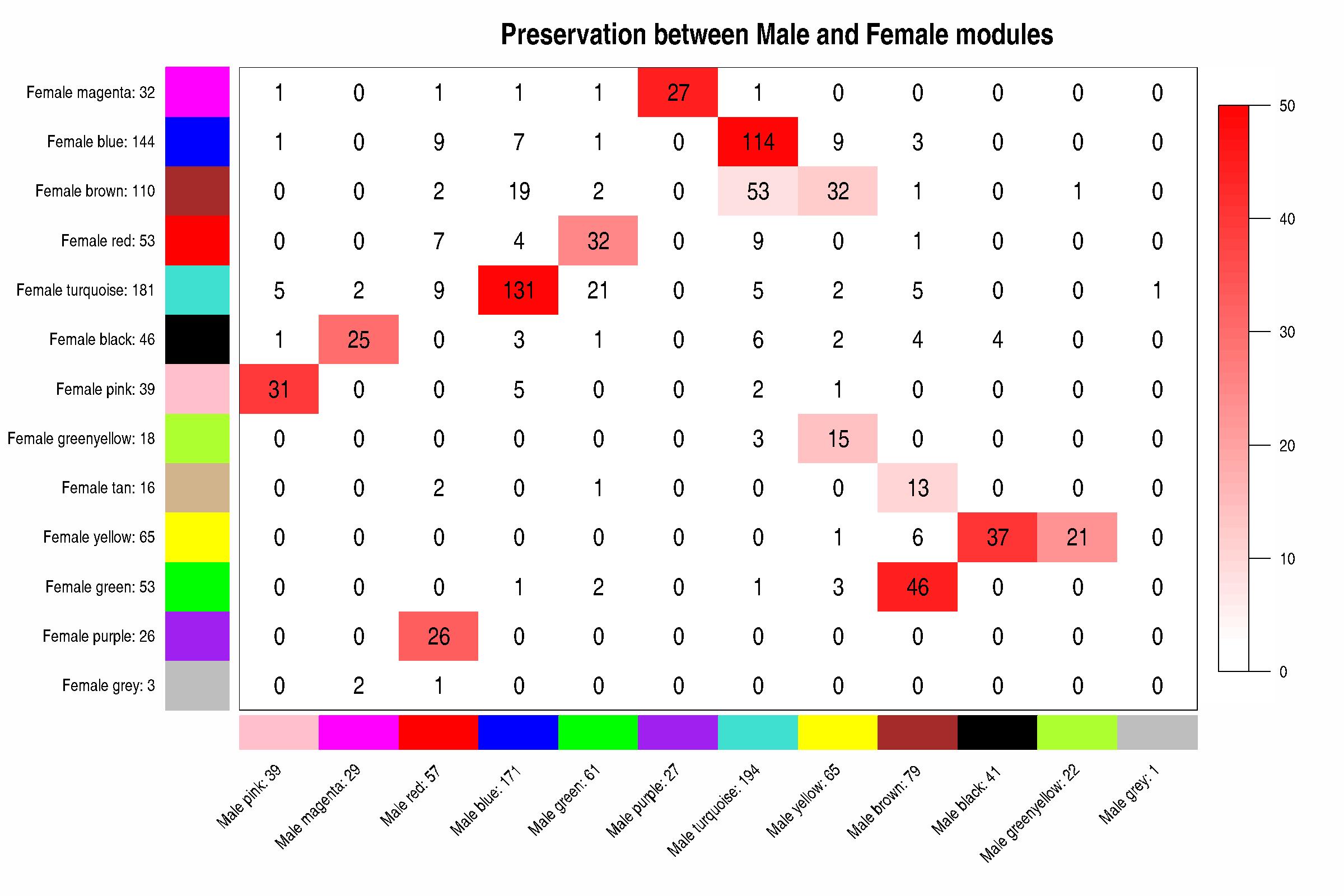


**Supplemental Figure S7: Correspondence of female set-specific modules and male set-specific modules in *SPIROMICS*.** Each row of the table corresponds to one male set-specific module (labeled by color as well as text), and each column corresponds to one female set-specific module. Numbers in the table indicate metabolite counts in the intersection of the corresponding modules. Coloring of the table encodes−log(p), with p being the Fisher’s exact test p-value for the overlap of the two modules. The stronger the red color, the more significant the overlap is.


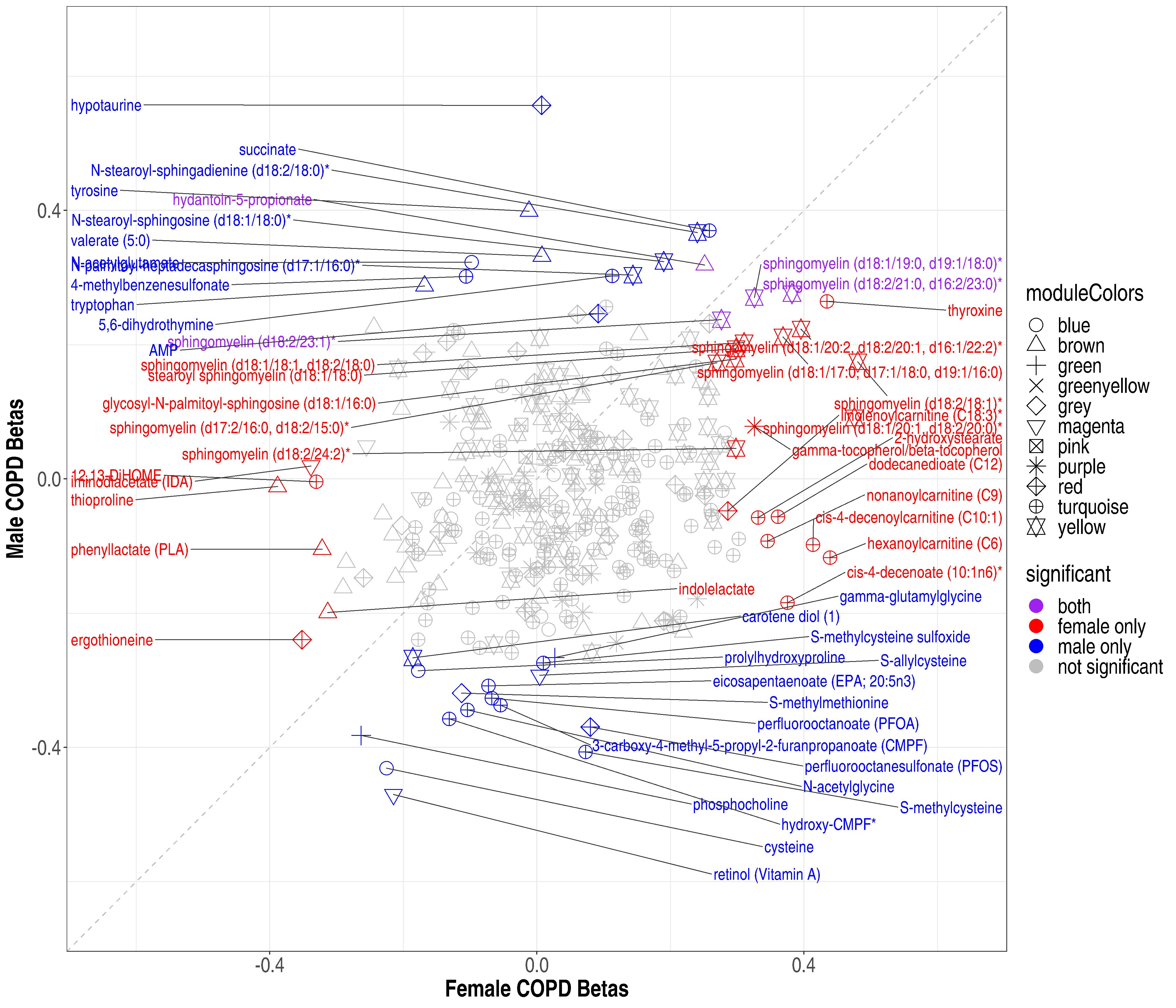


**Supplemental Figure S8: Scatter plot of sex specific betas for COPD models in *SPRIOMICS*.** The x-axis represents the beta estimates in the female stratum while the y-axis represents the beta estimates in the male stratum. Point shape corresponds with module assignment. Points are colored by significance in specific strata.
